# Social Determinants of Health and Long-Term Mortality of Patients with Chronic Subdural Hematoma: Is There an Association?

**DOI:** 10.3390/healthcare12161627

**Published:** 2024-08-15

**Authors:** Sanja Lepić, Aleksa Mićić, Milan Lepić, Lukas Rasulić, Stefan Mandić-Rajčević

**Affiliations:** 1 Faculty of Medicine, University of Belgrade, 11000, Belgrade, Serbia; sanja.lepic@vma.mod.gov.rs (S.L.); aleksamicic.md@gmail.com (A.M.); lukas.rasulic@gmail.com (L.R.); 2 Institute of Hygiene, Military Medical Academy, 11000 Belgrade, Serbia; 3 Clinic for Neurosurgery, University Clinical Center of Serbia, 11000 Belgrade, Serbia; 4 Medical Faculty of the Military Medical Academy, University of Defence, 11000 Belgrade, Serbia; milan.lepic@vma.mod.gov.rs; 5 Clinic for Neurosurgery, Military Medical Academy, 11000 Belgrade, Serbia; 6 School of Public Health and Health Management, University of Belgrade, 11000 Belgrade, Serbia; 7 Institute of Social Medicine, University of Belgrade, 11000 Belgrade, Serbia

**Keywords:** social determinants of health, chronic subdural hematoma, insurance, sentinel event, social support

## Abstract

(1) Background: A chronic subdural hematoma (CSDH) is considered an acute life-threatening event that is easily treated surgically, but little is known about the longer-term mortality of these patients. The objective of this study was to evaluate the association of social determinants of health (SDoH) and the long-term mortality of patients with a chronic subdural hematoma. (2) Methods: This retrospective cohort study included 121 (88 male and 33 female) patients with a surgically treated unilateral or bilateral CSDH. Mortality was evaluated at 1, 2, 6, and 12 months after treatment. (3) Results: Most of the patients were >65 and retired, N = 96 (79.3%); of them, the majority presented with a neurological deficit, N = 71 (73.9%). Patients who lived alone more often had a neurological deficit, N = 57 (75.0%), compared to those who lived in communities, N = 25 (55.5%). Mortality at 1, 2, 6, and 12 months after surgery was 10.7%, 17.4%, 19.0%, and 45.5%, respectively, and there was a significant difference in the median age between the survival and deceased groups at 1 month (*p* < 0.01), 2 months (*p* < 0.01), and 6 months (*p* < 0.01) of follow-up, but not in the long-term (12 months) follow-up (*p* = 0.200). Patients who lived alone had 3.7 times higher odds of dying at the 12-month follow-up (*p* < 0.01), compared to those who lived in the community. (4) Conclusions: Living alone is related to an increased case fatality risk after CSDH surgery in the Serbian context. Social determinants of health can be associated with CSDH presentation and survival, indicating that further studies should include SDoH to obtain a deeper understanding of the occurrence, presentation, and outcomes of SDoH and propose additional preventive measures.

## 1. Introduction

A chronic subdural hematoma (CSDH), contrary to (traumatic) acute subdural hematomas, which usually present within 3 days, is a collection of blood and fluid surrounded by membranes that accumulates between the arachnoid and dura mater over weeks to months, sometimes preceded by mild trauma. Contemporary neuroscience considers a CSDH as a benign entity, with a surprisingly simple and effective surgical treatment, a high rate of successful recovery (sometimes in only hours after surgery), and a good prognosis [1,2]. However, the few existing studies on the long-term outcomes after a CSDH reveal significant excess mortality compared to the general population [3,4,5] and compromised long-term survival. Rauhala et al. suggested that this excess mortality is associated with patient-related factors and characteristics rather than the CSDH’s characteristics itself [4]. CSDHs could therefore indicate patients’ frailty and represent a sentinel health event [5], of which survivors represent a vulnerable group that requires long-term, comprehensive, and person-centered care.

Numerous studies have emphasized that not only personal (gender, age) but also socioeconomic variables (marital status, place of residence, education level, work status, income) could be associated with health outcomes. This was summarized by the World Health Organization (WHO) [6] and modified by Barton and Grant (2006), who developed an influential model of the main determinants of health, in which, at the core, are constitutional factors such as gender, age, and genetics; overlapping layers represent individual lifestyle factors, the community, and the local economy, followed by the broader ‘environmental’ determinants [7].

Previous studies have never considered the impact of social determinants of health on the outcomes of CSDH patients. Since a CSDH appears to be an acute presentation of a slowly developing disease in a vulnerable population, our objective was to assess the impact that social determinants of health may have on these patients, to better understand the prognosis of CSDHs, with regard to the Serbian socioeconomic environment [8].

## 2. Materials and Methods

This retrospective cohort study involved 121 patients with a unilateral or bilateral symptomatic CSDH, surgically treated at the Clinic for Neurosurgery of the Military Medical Academy, Belgrade, Serbia.

The medical records were reviewed for the patients’ age, gender, place of residence, type of health insurance, work status, presence of comorbidities, and premorbid neurological status.

Clinical evaluation included a symptom assessment, neurological status determination, and the grading of the Glasgow Coma Scale (GCS) and Markwalder’s Grading Scale (MGS) scores.

Determinants of health included demographic characteristics (gender, age, and presence of associated diseases), as well as socioeconomic factors including work status (employed/retired), insurance type (civil/military), the patient’s social support (living alone/living with other family members), and the place of residence (urban/rural). Rural residency in Serbia was considered when the population density was lower than 150 residents/km^2^.

Survival was evaluated at 1 and 2 months after surgical treatment (short-term outcome), at 6 months (mid-term outcome), and at 12 months (long-term outcome).

Results are presented as counts (%), means ± standard deviations, or medians (25th–75th percentile) depending on the data type and distribution. Groups are compared using parametric (*t* test, ANOVA) and nonparametric (chi-square, Mann–Whitney U test, Kruskal–Wallis test) tests. Binary logistic regression was performed to analyze the impact of factors (age, gender, place of residence, work status, type of health insurance, presence of associated diseases, and social support) on survival at 1, 2, 6, and 12 months after surgical treatment. All *p* values less than 0.05 were considered significant. All data were analyzed using SPSS 20.0 (IBM Corp. Released 2011. IBM SPSS Statistics for Windows, Version 20.0. Armonk, IBM Corp.: New York, NY, USA) and R 3.4.2. (R Foundation for Statistical Computing, Vienna, Austria) [9].

## 3. Results

The study included 88 male and 33 female patients, with a median age of 75 (38–95) (Table 1). Comorbidities were present in the vast majority of patients (84.3%).

**Figure 1:**
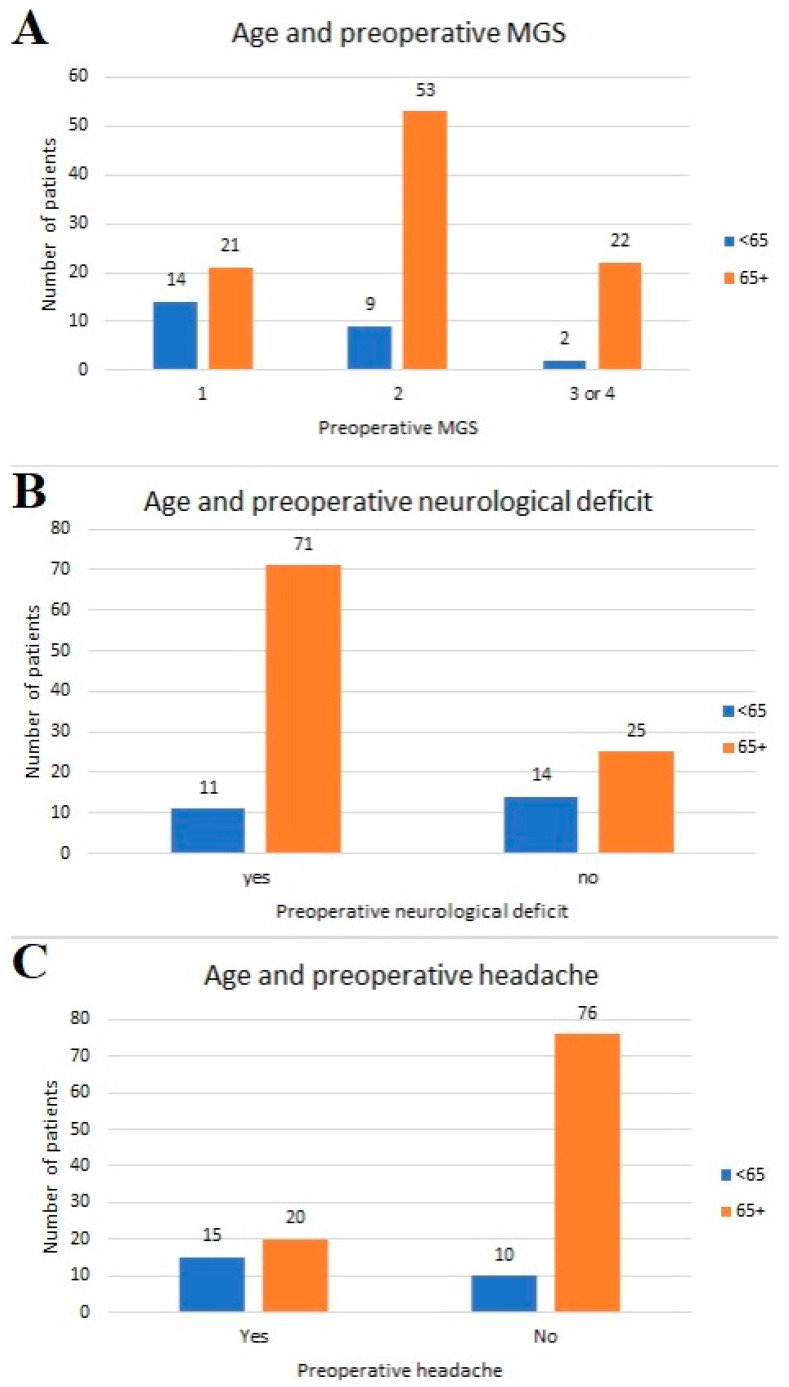
Distribution of patients in age groups with reference to (**A**) preoperative MGS; (**B**) preoperative neurological deficit; (**C**) presence of headache.

There was a significant difference in the age groups regarding preoperative MGS and the presence of a neurological deficit and headache (Figure 1). Most of the patients under 65 years of age (92%) had an MGS score of 2 or less, while most of the patients with preoperative neurological deficits (86.6%) and without headache (88.4%) were older than 65 years of age.

**Figure 2:**
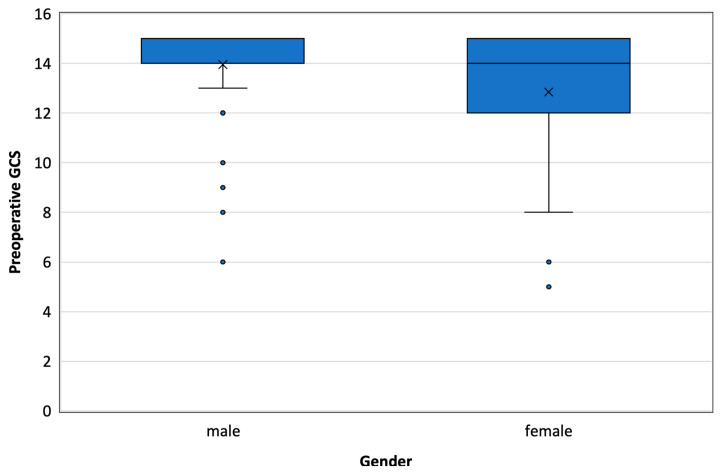
Box plot presenting the difference in the median GCS between males and females.

The median GCS was significantly lower in women compared to men (Figure 2). Comorbidities were more common in the groups with a higher MGS score (2) compared to the group that scored 1.

The majority of patients were retired—specifically, 99 (81.8%). The insurance category was predominantly civilian in 97 patients (80.2%), but the type of insurance being civilian or military was not associated with other factors or outcomes. Patients’ demographic, clinical, and socioeconomic features and the outcomes at the different stages of follow-up are presented in Table 2.

**Figure 3:**
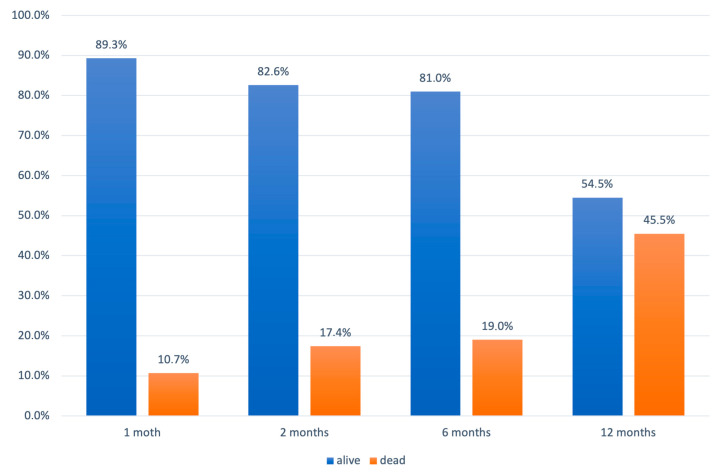
Distribution of the patients with respect to the outcomes at different follow-up points.

Mortality at 1, 2, 6, and 12 months after surgery was 10.7%, 17.4%, 19.0%, and 45.5%, respectively (Figure 3).

**Figure 4:**
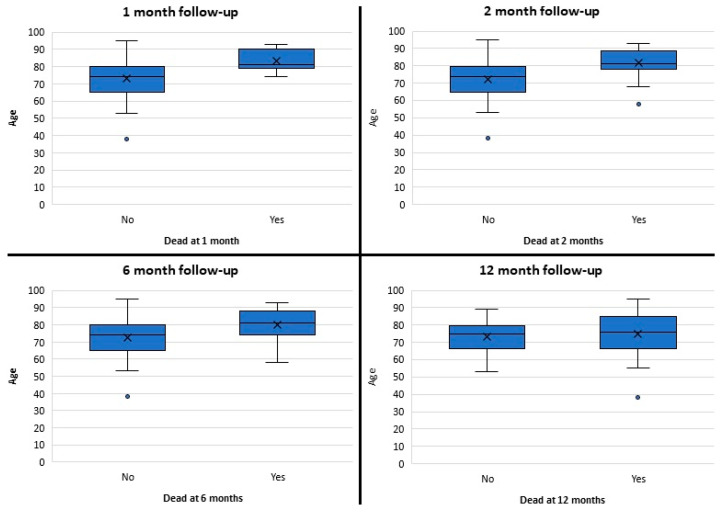
Box plots demonstrating patient age and outcomes at different periods of follow-up.

There was a significant difference in the median age between the survival and death groups at 1 month (U = 291, *p <* 0.01), 2 months (U = 483, *p <* 0.01), and 6 months (U = 622, *p <* 0.01) of follow-up (Figure 4). However, there were no significant differences at 12 months of follow-up (U = 1569, *p =* 0.200).

Binary logistic regression indicated that patients who lived alone had 3.7 times higher odds of dying at the 12-month follow-up (*p* < 0.01) compared to those who lived in the community.

## 4. Discussion

This study analyzed the associations between social determinants of health and long-term mortality from a chronic subdural hematoma. Our findings showed that the work status and health insurance type were not associated with long-term mortality. Age was associated with mortality after 1, 2, and 6 months of follow-up, but not at 12 months of follow-up. Finally, living alone, as a proxy for a lack of social support, was associated with 3.7 times higher odds of dying at 12-month follow-up.

A chronic subdural hematoma is a relatively common entity, especially in the elderly population [10]. Numerous risk factors were identified, such as the male gender, alcohol abuse, epilepsy, and the long-term use of antiaggregant, anticoagulant, or non-steroid anti-inflammatory drugs [4,11,12,13,14,15]. Although its name implies a traumatic origin, the exact etiopathogenesis of CSDHs remains unclear, and the initial “pachymeningitis hemorrhagica interna” (as named by Virchow in 1856) appears to better correspond to the recent findings and the complex nature of the CSDH [2,16].

Older patients with a chronic subdural hematoma (CSDH) often experience motor deficits and cognitive impairments, while younger patients commonly report headaches [17]. Our study aligns with these findings. Additionally, age plays a significant role in the short-term outcomes of CSDH treatment, with higher mortality rates observed in older age groups, particularly those older than 80 or 85 years [4,5]. However, our study suggests that while older age impacts short- and medium-term mortality, it does not significantly affect the long-term outcomes.

Gender differences in CSDH occurrence are noted, with males predominating, possibly due to their increased injury exposure or brain morphology [13,14,18]. Although gender may influence the treatment outcomes, the long-term results do not seem to be gender-associated, consistent with our study’s findings [19]. Some studies have found that consciousness disorder during the course of a CSDH was more common in women compared to men [20], and, according to another study, only in the group under 40 years of age [19]. However, with reference to Wang et al., there were no differences in consciousness disorder occurrence between genders [21]. Regarding the results of our study, women had a lower median GCS compared to men and comprised the majority of comatose patients. The difference in these results may be due to the already present cognitive decline in those reporting only when the state of consciousness was disturbed; however, no statistically significant relation was found in this study.

A neurological deficit, in our study, was significantly more common in patients who lived alone compared to those who lived in the community. This difference is most likely due to the community living being more critical regarding timely referral to medical care, rather than the patients themselves.

Previous studies have emphasized that the preoperative presence of comorbidities is associated with a worse postoperative outcome, of which dementia and alcoholism were the most significant for long-term mortality [4]. In our study, long-term mortality was not associated with the presence of comorbidities or with the presence of alcoholism and/or dementia; however, only a few of our patients without comorbidities died during the follow-up period. This fact implies the importance of comorbidities in determining the outcomes, highlighting the majority of frail people in this group of patients.

According to the literature, poor social support is associated with higher mortality [22,23]. However, there are no studies on the impact of living alone, as a proxy for social support, on the outcome after CSDH surgery. In the Serbian environment, the relationship between living alone and the outcomes of CSDHs appears significant. While elderly individuals who live alone are at a higher risk of delayed diagnosis and treatment for CSDHs, often due to the lack of immediate assistance and regular monitoring, prolonged recovery times and higher morbidity rates, socioeconomic factors, and limited access to healthcare services in certain areas can exacerbate the outcomes [8]. Therefore, living alone is a critical factor influencing the management and prognosis of CSDHs in Serbia, highlighting the need for improved support systems and healthcare accessibility for these vulnerable individuals.

As expected, and in accordance with previous studies, the long-term mortality in our study was notably higher than the short- and mid-term mortality [3,4]. These results indicate that long-term survival after a CSDH diagnosis is poor and that CSDHs could be considered a sentinel health event, as proposed by Dumont et al. [5]. Despite the simple surgical treatment and successful postoperative withdrawal of symptoms, a large proportion of patients have a high risk of death in the first year after a CSDH. The occurrence of a CSDH should direct the attention of the family and social institutions to improve the survival prognosis of these patients. As with hip fractures [24], CSDHs, as a sentinel health event [5], are not specific (like a transitory ischemic attack or chest pain) [25] but general and exhibit a wider spectrum of health impairments and thus place a heavy burden on health systems and social institutions.

Potential interventions and policies should start with the recognition of CSDHs as a separate entity from a traumatic or spontaneous subdural hemorrhage in future disease classifications and as a sentinel health event. These could be followed by targeted healthcare policies for at-risk patients, focusing on quaternary prevention (the prevention of the overuse of anticoagulation and antiaggregation dugs and other risk factors), community support systems such as home visits for patients living alone, and enhanced access to healthcare resources (similar to cancer or diabetes patients) due to their frailty.

The main limitations of this study include the small cohort of patients, its retrospective nature, and the fact that the study was based on medical records, which limited the information retrieval and the number of social determinants of health that we could study. Furthermore, to perform a survival analysis as a more robust method, it is necessary to collect exact data about the months of survival, instead of verifying the survival in fixed intervals. Therefore, future studies should focus on a broader range of social determinants of health, as proposed by the WHO, and collect the relevant information, including the exact survival in months, in a prospective multi-center study, possibly extending the period of follow-up to 2 years to better understand the mortality/survival after a CSDH and the factors associated with it.

## 5. Conclusions

A CSDH represents a common acute event in the elderly, which is easily surgically treated but continues to pose an additional burden and leaves the patient with a higher risk of death, even after one year of successful recovery. Living alone is associated with a higher risk of mortality in patients after CSDH surgery in the Serbian environment. Understanding the association between social determinants of health and long-term CSDH outcomes could have prognostic value in the prediction of the long-term mortality of patients after CSDH surgery; however, future studies with larger cohorts should confirm these findings.

## Figures and Tables

**Table 1:** Distribution of patients according to health determinants and preoperative neurological status.

Health Determinants	Preoperative Neurological Status									
	Neurological Deficit			Headache			MGS ^1^			
(n = 121)	Yes	No	*p* Value	Yes	No	*p* Value	1	2	≥3	*p* Value
Age			<0.01			<0.01				<0.01
<65 years	11	14		15	10		14	9	2	
>65 years	71	25		20	76		21	53	22	
Gender			0.874			0.487				0.432
Male	60	28		27	61		27	46	15	
Female	22	11		8	25		8	16	9	
Place of residence			0.325			0.022				0.908
Urban	68	35		34	69		30	52	21	
Rural	14	4		1	17		5	10	3	
Presence of associated disease			0.124			<0.01				<0.01
Yes	72	30		20	82		23	57	22	
No	30	9		15	4		12	5	2	
Living alone			0.027			0.216				0.067
Yes	57	19		19	57		20	36	20	
No	25	20		16	29		15	26	4	
Health insurance			0.537			0.977				0.456
Civil	67	30		28	69		30	47	20	
Military	15	9		7	17		5	15	4	
Work status			<0.01			<0.01				<0.01
Employed	9	13		14	8		13	8	1	
Retired	73	26		21	78		22	54	23	

^1^ Markwalder Grading Scale.

**Table 2:** Distribution of the patients according to the health determinants and the outcomes at the different stages of follow-up.

N of Patients = 121		Total	1 Month			2 Months			6 Months			12 Months			Total Mortality (%)
			Alive	Dead	Mortality (%)	Alive	Dead	Mortality (%)	Alive	Dead	Mortality (%)	Alive	Dead	Mortality (%)	
Gender	Male	88	78	10	10 (24%)	71	17	7 (17%)	71	17	/	46	42	25 (60%)	42 (47%)
	Female	33	30	3	3 (23%)	29	4	1 (8%)	27	6	2 (15%)	20	13	7 (54%)	13 (39%)
Place of residence	Urban	103	93	10	10 (21%)	86	17	7 (15%)	84	19	2 (4%)	56	47	28 (60%)	47 (42%)
	Rural	18	15	3	3 (38%)	14	4	1 (13%)	14	4	/	10	8	4 (50%)	8 (44%)
Work status	Employed	22	22	0	0 (0%)	21	1	1 (11%)	20	2	1 (11%)	13	9	7 (78%)	9 (41%)
	Retired	99	86	13	13 (28%)	79	20	7 (15%)	78	21	1 (2%)	53	46	25 (54%)	46 (46%)
Insurance	Civil	97	88	9	9 (20%)	80	17	8 (18%)	78	19	2 (4%)	52	45	26 (58%)	45 (46%)
	Military	24	20	4	4 (40%)	20	4	/	20	4	/	14	10	6 (60%)	10 (42%)
Associated diseases	Yes	102	89	13	13 (27%)	81	21	8 (16%)	79	23	2 (4%)	53	49	26 (53%)	49 (48%)
	No	19	19	0	/	19	0	/	19	0	/	13	6	6 (100%)	6 (32%)
Alcohol abuse or dementia	Yes	17	11	6	6 (100%)	11	6	/	11	6	/	11	6	/	6 (35%)
	No	104	97	7	7 (14%)	89	15	8 (16%)	87	17	2 (4%)	55	49	32 (65%)	49 (47%)
Living	Alone	76	67	9	9 (21%)	60	16	7 (17%)	59	17	1 (2%)	42	34	25 (60%)	42 (55%)
	With someone	45	41	4	4 (31%)	40	5	1 (8%)	39	6	1 (8%)	42	13	7 (54%)	13 (29%)

## Data Availability

The data presented in this study are available on request from the corresponding author, being a part of a larger dataset.
